# A Screening Pipeline for Antiparasitic Agents Targeting *Cryptosporidium* Inosine Monophosphate Dehydrogenase

**DOI:** 10.1371/journal.pntd.0000794

**Published:** 2010-08-10

**Authors:** Lisa Sharling, Xiaoping Liu, Deviprasad R. Gollapalli, Sushil K. Maurya, Lizbeth Hedstrom, Boris Striepen

**Affiliations:** 1 Center for Tropical and Emerging Global Diseases, University of Georgia, Athens, Georgia, United States of America; 2 Department of Biochemistry, Brandeis University, Waltham, Massachusetts, United States of America; 3 Department of Biology, Brandeis University, Waltham, Massachusetts, United States of America; 4 Department of Chemistry, Brandeis University, Waltham, Massachusetts, United States of America; 5 Department of Cellular Biology, University of Georgia, Athens, Georgia, United States of America; George Washington University, United States of America

## Abstract

**Background:**

The protozoan parasite *Cryptosporidium parvum* is responsible for significant disease burden among children in developing countries. In addition Cryptosporidiosis can result in chronic and life-threatening enteritis in AIDS patients, and the currently available drugs lack efficacy in treating these severe conditions. The discovery and development of novel anti-cryptosporidial therapeutics has been hampered by the poor experimental tractability of this pathogen. While the genome sequencing effort has identified several intriguing new targets including a unique inosine monophosphate dehydrogenase (IMPDH), pursuing these targets and testing inhibitors has been frustratingly difficult.

**Methodology and Principal Findings:**

Here we have developed a pipeline of tools to accelerate the *in vivo* screening of inhibitors of *C. parvum* IMPDH. We have genetically engineered the related parasite *Toxoplasma gondii* to serve as a model of *C. parvum* infection as the first screen. This assay provides crucial target validation and a large signal window that is currently not possible in assays involving *C. parvum*. To further develop compounds that pass this first filter, we established a fluorescence-based assay of host cell proliferation, and a *C. parvum* growth assay that utilizes automated high-content imaging analysis for enhanced throughput.

**Conclusions and Significance:**

We have used these assays to evaluate *C. parvum* IMPDH inhibitors emerging from our ongoing medicinal chemistry effort and have identified a subset of 1,2,3-triazole ethers that exhibit excellent *in vivo* selectivity in the *T. gondii* model and improved anti-cryptosporidial activity.

## Author Summary

Persistent diarrhea is a leading cause of illness and death among impoverished children, and a growing share of this disease burden can be attributed to the parasite *Cryptosporidium*. There are no vaccines to prevent *Cryptosporidium* infection, and the treatment options are limited and unreliable. Critically, no effective treatment exists for children or adults suffering from AIDS. *Cryptosporidium* presents many technical obstacles for drug discovery; perhaps the most important roadblock is the difficulty of monitoring drug action. Here we have developed a set of methods to accelerate the drug discovery process for cryptosporidiosis. We exploit the opportunities for experimental manipulation in the related parasite *Toxoplasma* to genetically engineer a *Cryptosporidium* model. This new model parasite mirrors the metabolism of *Cryptosporidium* for a particularly promising drug target that supplies the building blocks for DNA and RNA. Drug effectiveness can be assayed through simple fluorescence measurements for many candidates. Using this assay as an initial filter, and adapting other assays to a high throughput format, we identify several novel chemical compounds that exhibit markedly improved anti-cryptosporidial activity and excellent selectivity.

## Introduction

Gastrointestinal diseases remain the largest threat to the health of infants and small children in environments with low income and poor sanitation. While acute diarrheal disease claims numerous lives, chronic or recurrent forms can result in stunting of physical and intellectual growth in an even larger number of children. The aetiology of diarrheal disease in children is complex, involving a large group of viral, bacterial, protozoan and metazoan pathogens. Among these the protozoan parasites, *Cryptosporidium parum* and *hominis* are epidemiologically important pathogens [1], [2].

*Cryptosporidium* causes acute self-limiting gastrointestinal disease in healthy individuals. Immunity is slow to develop and the disease can be recurrent and protracted in malnourished children [3]–[6]. Malnourished children are not only more susceptible to severe cryptosporidiosis, but the disease itself is an important contributing factor to malnutrition [7]. In immunocompromised individuals like those suffering from AIDS, cryptosporidiosis is a chronic and life-threatening disease [8]. The persistent and resilient nature of the infective oocyst stage in drinking and recreational water poses significant challenges for controlling transmission even in industrialised nations. No vaccines exist, and the currently available drugs are inadequate. The more widely used drugs paromomycin and azithromycin are unreliable and the efficacy of nitazoxanide, which recently received FDA approval, is dependent upon a robust immune response [9]. The options in particular for the treatment of chronic AIDS-related cryptosporidiosis are severely limited [10] and there is an overall urgent need for new chemotherapy.

**Figure 1:**
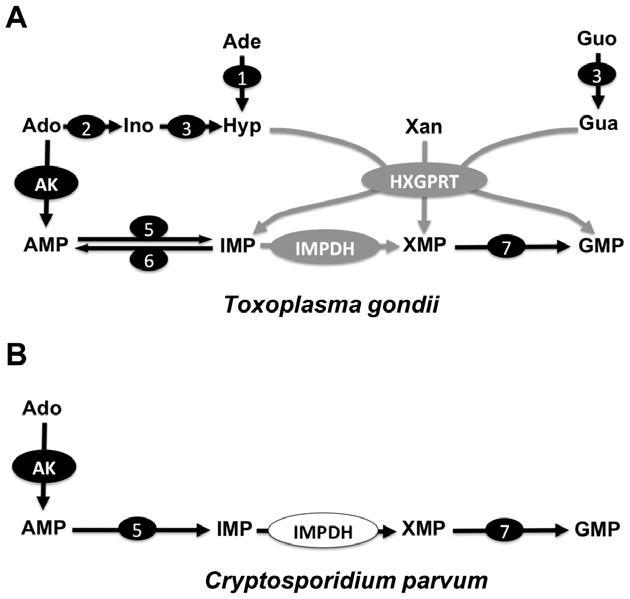
Schematic of purine salvage in *T. gondii* and *C. parvum*. **A**, Genetic studies have shown that the salvage of adenosine via adenosine kinase is the predominant route to GMP for *T. gondii* [59] and IMPDH catalyses the rate limiting step of this pathway. However in the absence of adenosine kinase, *Tg*HXGPRT allows for the salvage of adenosine, adenine and guanosine such that the activity of *Tg*HXGPRT is sufficient for parasite proliferation [59]. Several transporters for the uptake of nucleobases [60] and nucleotides have been characterised in *T. gondii* [60]–[62]; **B**) Unlike *T. gondii* and other Apicomplexa, *C. parvum* lacks HXGPRT [12], [13] and is dependent on the salvage of adenosine and thus the activity of *Cp*IMPDH. A single adenosine transporter has been identified in the genome of *C. parvum* [12], [59]. The *T. gondii* pathways shown in grey in **A** highlight the genes disrupted in the parasite clones used in this study, *TgHXGRT* in a previous studies (HXGPRT;[41]) and *TgIMPDH* in this study. Hyp, hypoxanthine; Xan, xanthine; Gua, guanine; Guo, guanosine; Ade, adenine; Ado, adenosine; Ino, inosine; AMP, adenosine monophosphate; IMP, inosine monophosphate; XMP, xanthosine monophosphate; GMP, guanosine monophosphate; HXGPRT, hypoxanthine xanthine gunanine phosphoribosyltransferase; IMPDH, IMP dehydrogenase, 1, adenine deaminase; 2, adenosine deaminase; 3, purine nucleoside phosphorylase; 4, adenosine kinase; 5, AMP deaminase; 6, adenoylsuccinate synthase and adenoylsuccinate lyase; 7, GMP synthase [13], [59], [63].

The sequencing of the genomes of Cryp*tosporidium parvum* and *hominis* revealed a highly streamlined anabolic metabolism with potential choke points that might be exploited in drug design [11], [12]. One such vulnerability lies in the pathway that supplies purine nucleotides for the synthesis of DNA and RNA. Like all protozoan parasites, *Cryptosporidium* is incapable of *de novo* purine synthesis and relies on salvage of purines from the host. While many parasites, including the related Apicomplexa *Toxoplasma* and *Plasmodium*, use hypoxanthine-xanthine-guanine phosphoribosyltransferase (HXGPRT, Figure 1) to salvage a broad range of purine bases this enzyme is missing in *Cryptosporidium* [12], [13]. Instead, *Cryptosporidium* relies solely on the salvage of adenosine to provide both adenine and guanine nucleotides. This simplified pathway is initiated by adenosine kinase and critically depends on the activity of inosine monophosphate dehydrogenase (*Cp*IMPDH). Surprisingly, the *Cp*IMPDH gene appears to have been acquired through lateral gene transfer from an ε-proteobacterium [13], [14]. Detailed kinetic analysis of this prokaryote-like enzyme demonstrated that *Cp*IMPDH is very different from its human homologs [13], [15]. The “drugability” of IMPDH is well established as inhibitors of human IMPDHs have been used clinically as immunosuppressants as well as for the treatment of viral infections and cancer [16]–[18]. Taken together, these observations suggest that *Cp*IMPDH is a promising therapeutic target for cryptosporidiosis [13]–[15], [19].

We have recently discovered several new classes of selective inhibitors of *Cp*IMPDH in a high throughput screen [20]. Extensive medicinal chemistry optimization has produced nanomolar inhibitors with greater than 10^3^-fold selectivity for *Cp*IMPDH over the human enzymes [21], [22]. Further elaboration of these compounds requires a rapid and reliable assay to evaluate their anti-parasitic activity. The screening of compounds against *C. parvum* has proven challenging and the main roadblock in our drug development program. This is partly because the parasite cannot be cultured continuously in tissue culture. Although several growth assays have been developed, including real-time PCR protocols [23]–[28], immunoassays [29], [30], and image analysis routines [31], these assays are labour intensive and expensive, or are not amenable to higher throughput. The scarcity of experimental tools for *C. parvum* also makes it difficult to investigate the mechanism of inhibitor action.

In this study we describe an assay pipeline to evaluate the anti-cryptosporidial activity of *Cp*IMPDH inhibitors in tissue culture models. We have genetically engineered the related apicomplexan parasite *T. gondii* to mirror the purine pathway of *Cryptosporidium* and to depend on *Cp*IMPDH for survival. This system provides a rapid assessment of anti-parasitic potential and a quantitative read of target specificity. To further evaluate if anti-cryptosporidial activity arises from off-target effects on the host cell, we devised a simple and highly sensitive host cell growth assay using an HCT-8 line engineered to express a yellow fluorescent protein. Lastly, we have developed a new high-content imaging assay to measure *C. parvum* proliferation directly. We have utilized these assays to probe the structure-activity relationship for a series of *Cp*IMPDH inhibitors [20], [22]. We discovered five compounds that exhibited potency and selectivity in the *T. gondii* model, including two that show sub-micromolar anti-cryptosporidial activity.

## Materials and Methods

### Cosmid modification

Cosmid TOXOU05 [32] was selected using the ToxoDB database [33]. The 42.2 Kb genomic DNA insert of TOXTOU05 spans the *Tg*IMPDH open reading frame with 14.3 Kb and 22.7 Kb of flanking sequence at the 5′ and 3′ ends respectively. A schematic overview of the cosmid modification steps is shown in Figure S1 [34]. Initial cosmid amplification was performed in *E. coli* strain XL1Blue using LB containing 10 µg/ml kanamycin. The QIAprep Spin Miniprep kit (QIAGEN) was used with slight modification to the standard protocol for cosmid purification. Following the addition of buffer P3 and centrifugation cosmid DNA was directly precipitated from the supernatant using 0.7 volumes of isopropanol. Cosmid DNA (200 ng) was introduced into the *E. coli* EL250 strain [35] by electroporation (ECM 630, 1 mm cuvette, 1.75 kV, 250 Ω, and 25 µF; BTX; Holliston, MA) and bacteria were propagated in LB (50 µg/ml kanamycin) and rendered electro-competent. A cassette, comprising a bacterial gentamycin selectable marker and the chloramphenicol resisftance gene flanked by the 5′ and 3′ UTRs of the *T. gondii* GRA1 gene (for selection in the parasite) was amplified from plasmid piCG [34] by PCR. To drive recombination between the cosmid and the GENTgraCATgra cassette to excise the IMPDH coding sequence, ∼50 bp overhangs homologous to the sequence immediately flanking the start and stop codons of the *Tg*IMPDH gene were included in the primers (primers 973: 5- (CCTGCGTCGTTTTTGCCTCGTGGCTGTTTGGCTTGTGGGTGTCTTTCCACATACGACTCACTATAGGGCGAATTGG) and 923: 5′- GTTCCCCTTTTTATATTTCTTCTTCCGTTGCACTTCCCCAGCGAAACAAAATACGACTCACTATAGGGCGAATTGG). The PCR amplified cassette was gel purified and 200 ng were electroporated into the EL250 strain [35] bearing the TOXO05 parent cosmid and bacteria were selected using kanamycin (50 µg/ml) and gentamycin (10 µg/ml). To recycle the bacterial gentamycin selectable marker, the sequence was removed from the modified cosmid using flanking Flp sites and induction of flipase [34]. Cosmid DNA was isolated from kanamycin resistant, gentamycin sensitive clones and the removal of the gentamycin sequence was confirmed by PCR. To exchange the parasite selectable marker DHFR with the fluorescent marker YFYFP the tubYFPYFPmicGENT expression cassette was recombineered into the TOXOU05graCATgraΔIMPDH cosmid in EL250 *E. coli* following the method described above. The tubYFPYFPmicGENT cassette was amplified from the pYG plasmid using primers (874: 5′-GTTCCCCTTTTTATATTTCTTCTTCCGTTGCACTTCCCCAGCGAAACAAAATACGACTCACTATAGGGCGAATTGG and 875: 5′- GTCGTGGGCAGACAGCAACAGTCCAGCACTCTAGCGGCATACAGAACGATCTTCTTAGGTGGCGGTACTTGGGTC). Approximately 50 bp overhangs homologous to the sequence flanking the DHFR coding sequence were included in these primers to drive recombination to create TOXOU05CATΔIMPDHYFPYFP. Restriction enzymes HindIII, NdeI and NotI were used to confirm appropriate recombination in the final cosmid DNA preparation.

### Targeting the *Tg*IMPDH locus

*T. gondii* RHΔHX-CpIMPDH-5MX clone [14] was electroporated with 25 µg of circular TOXOU05CATΔIMPDHYFPYFP cosmid (Figure S1). The parasites were cultured in hTERT cells and selected under 6.8 µg/ml chloramphenicol as described previously [36]. Chloramphenicol resistant parasite clones were PCR screened using 2 primer pairs to the graCATgra cassette and 2 primer pairs to the *Tg*IMPDH coding sequence and a single primer pair to the tubYFPYFPmicGENT cassette. This PCR screen indicated that double-homologous recombination at the *Tg*IMPDH locus had occurred in 2 clones. Southern blotting confirmed that double homologous recombination had occurred to replace the native *Tg*IMPDH locus with the graCATgra expression. Probe 1 was amplified from wild-type *T. gondii* genomic DNA using primers 1108 (5′-CGATGTCGGGGAATCACAAGCGAGAACA) and 1109 (5′-ATGTCTTCTACGAARGCGRGAGTGGTGC).

### Isolation of fluorescent *T. gondii* parasite and fluorescent growth assay

Wild-type *T. gondii* and *T. gondii*-ΔHXGPRT parasites were transfected with plasmid ptubYFPYFsagCAT [37] which drives constitutive cytoplasmic expression of two copies of the yellow fluorescent protein. Similarly, plasmid pCTR2T [38] was transfected into *T. gondii*-*Cp*IMPDH resulting in constitutive cytoplasmic expression of a tandem of the tomato variant of the red fluorescent protein [39]. Fluorescent parasites were selected and cloned by cell sorting using a MoFlo Legacy instrument (Beckman Coulter, Inc). Growth of fluorescent clones was monitored in 96 well plates as previously described [37]. Briefly, fluorescence was read daily with a SpectraMax M22/M2e (Molecular Devices) plate reader (Ex 485, Em 530) for 6–7 days. For each of the three fluorescent *T. gondii* clones percent growth inhibition was calculated on a day within the exponential phase of growth for the purpose of determining an IC_50_ for each compound.

### Southern blotting

*T. gondii* genomic DNA was extracted using the DNeasy kit (QIAGEN). 1.0 µg of genomic DNA and 2.5 ng of cosmid DNA was digested for 14 hours with Xho1 and separated on a 0.9% TBE agarose gel and transferred overnight by downward transfer. The blot was hybridized over night with probe 1, which was amplified from wild-type *T. gondii* genomic DNA using primers 1108 (5′-CGATGTCGGGGAATCACAAGCGAGAACA) and 1109 (5′-ATGTCTTCTACGAARGCGRGAGTGGTGC).

### *In vitro* culture of *Cryptosporidium parvum*

The human ileocecal adenocarcinoma epithelial cell line, HCT-8, was used to support *C. parvum* infection *in vitro*. HCT-8 cells were maintained in RPMI-1640 (Hyclone) supplemented with 10% FBS, 1 mM sodium pyruvate, 50 U/m penicillin, 50 µg/ml streptomycin, and amphotericin B. *Cryptosporidium parvum* oocysts were a kind gift from either Dr. Mead (Emory University) or Dr. Kissinger (University of Georgia). HCT-8 cells (2×10^5^) were seeded into black, optical quality, thin bottom, 96-well plates (DB Falcon) to achieve a 70% confluent monolayer on the day of infection. To facilitate oocyst excystation, a procedure described by Gut *et al.*, was followed. Briefly, oocysts were washed twice with 1 ml of PBS (pH 7.2), incubated for 10 minutes at 37°C in 1 ml 10 mM of HCl and then incubated for a further 10 minutes in 0.2 ml of 200 µM sodium taurocholate at 15°C [40]. This oocyst suspension was diluted directly with DMEM (Hyclone) supplemented with 2% FBS, 50 U/ml penicillin, 50 µg/ml streptomycin, amphotericin B and 0.2 mM L-glutamine (infection medium) to inoculate host cell monolayers at 5×10^5^ oocysts per well. Host cell monolayers were incubated for 3 hours at 37°C prior to removal of unexcysted oocysts by aspiration and PBS wash. Infection medium was then added to the monolayers and infection was allowed to progress for 48 hours.

### *Vicia villosa* lectin (VVL) immunofluorescence assay and high content imaging

The VVL IFA was performed in a 96-well format. Following 48 hours of culture *C. parvum* infected HCT-8 cells were washed with PBS and fixed with 0.2 ml/well of 3% paraformaldehyde/PBS, permeabilized with 0.25% Triton-X-100/PBS and blocked with 4% BSA/PBS. 0.1 ml of fluorescein (FITC)-conjugated VVL (Vector Labs 0.5 µg/ml) in 1% BSA/PBS was applied to wells and incubated for 45 minutes. The plates were washed twice with 200 µl/well of PBS. In the first wash DAPI was included at 0.1 µg/ml. Finally 200 µl/well of PBS was added to the plates prior to storage at 4°C protected from light.

Confocal images were acquired using a BD Biosciences Bioimager P435. The instrument was programmed to automatically change position and focus, capture montage images of 9–16, 40× fields per well, and then compress and save the image files. Object finding algorithms were developed for the recognition of FITC-VVL labelled *C. parvum* parasites and DAPI labelled host cell nuclei using Attovision Software (BD Biosciences). Briefly, to identify FITC-VVL labelled parasites, the object finding algorithm included, a flat field correction, a polygon shape setting, a 3×3 rolling ball filter, manual pixel intensity thresholding (100 min – 4095 max), and minimum and maximum object size cut-offs of 5 pixels and 350 pixels respectively. The algorithm optimised for finding DAPI labelled host cell nuclei also included flat field correction and polygon shape setting, but applied a 25×25 rolling ball filter which is more suitable for larger objects, an automated pixel intensity threshold, minimum and maximum object size cut-offs of 200 pixels and 4000 pixels respectively, and a watershed step with an erosion factor of 8 to aid in the separation of adjacent densely packed nuclei. Note that the intensity and area size cut off exclude detection of the much smaller parasite nuclei (the human genome is three hundred times larger than the *C. parvum* genome).

The object finding analysis steps described above recorded the number and area of objects per montage image. The output files along with a plate map file were then passed to an automated analysis pipeline (Pipeline Pilot Software, Accelrys) to calculate the mean number of parasites, the ratio of parasite number to host cell nuclei number, the mean area of parasites and host cell nuclei by well, and the percentage growth by treatment as compared to the no drug control. These analyses were exported in graphical format for each plate and the numerical values tabulated in html format.

All test compounds were stored as 0.1 M stocks in DMSO at −20°C and further diluted in DMSO to a 200× working stocks for each dilution, such that the final concentration of DMSO in the infection medium was uniformly 0.5%. For the no drug control, DMSO alone was added to triplicate wells. As a control, a high paromomycin concentration (0.8 mg/ml) was included on each plate in triplicate wells. Plates where this paromomycin control did not inhibit 70–80% of parasite growth were manually inspected and omitted from the final analysis. To control for potential false-positive parasite detection uninfected wells were scored in each plate and the typically low background counts were automatically subtracted from the test wells.

### Fluorescent HCT-8 host cell growth assay

HCT-8 cells were transfected with plasmid pmaxGFP (Amaxa) using Lipofectamine (Invitrogen). Fluorescent lines were then selected and cloned using cell sorting. Confluent monolayers of pmaxGFP expressing cells were harvested from T75 flasks and passed through a 40 µm cell strainer. Cells were seeded at 4000 cells per well into black, optical quality, thin bottom, 96-well plates. All test compounds were diluted in DMSO as described. The fluorescence was read daily with a SpectraMax M22/M2e (Molecular Devices) plate reader (Ex 485, Em 530) for 6–7 days. Percent inhibition was calculated on a day within the exponential phase of growth.

### Compound synthesis

All compounds were synthesized using procedures previously described [22]. Compounds A61, A64, A68 and A99 were not previously described. The NMR spectra for these compounds are included as Supplemental Materials S1.

## Results and Discussion

### Engineering a *Toxoplasma* reporter parasite suitable for the screening of *Cp*IMPDH inhibitors

**Figure 2:**
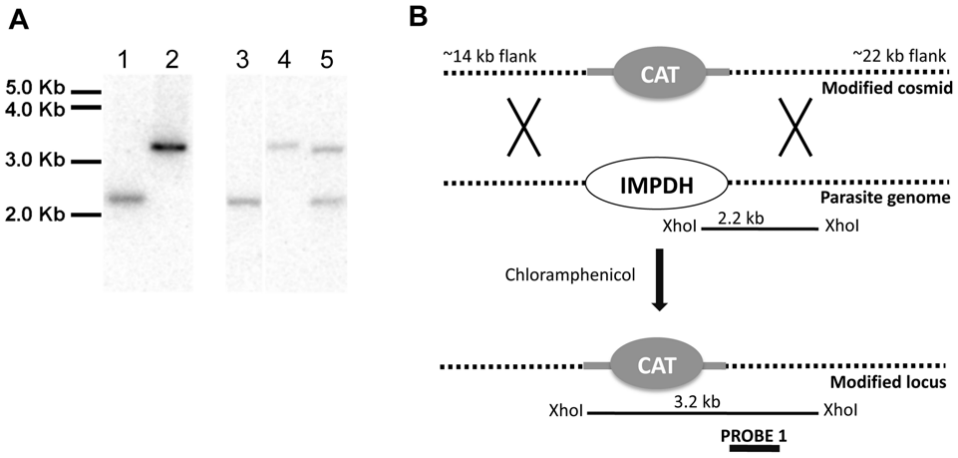
Disruption of *Tg*IMPDH to generate a *T. gondii* parasite dependent on *Cp*IMPDH. **A** shows a Southern blot confirming the replacement of the native *Tg*IMPDH coding sequence. Genomic parasite DNA or purified cosmid DNA was digested with XhoI, subjected to electrophoresis and blotting, and hybridized with a probe specific to the region shown in **B**. In **A**, lane 1 shows the wild-type cosmid TOXOU05, lane 2 the knock-out cosmid TOXOU05CATΔIMPDHYFPYFP, lane 3 the parent parasite *T. gondii* RHΔHX-CpIMPDH-5MX, lane 4 the *T. gondii*-*Cp*IMPDH-ΔHXGPRT-Δ*Tg*IMPDH knock-out parasite, and lane 5 a merodiploid transformant that retained the native *Tg*IMPDH locus. Note that *T. gondii*-*Cp*IMPDH-ΔHXGPRT-Δ*Tg*IMPDH is hereafter referred to as *T. gondii*-*Cp*IMPDH.

To facilitate screening of antiparasitic activity, we constructed a *T. gondii* reporter parasite that mirrors the *Cryptosporidium* purine metabolism. Figure 1 summarizes the main differences between the two parasites in this pathway. We started with a *T. gondii* knockout mutant that, like *C. parvum*, lacks the ability to salvage xanthine and guanine via HXGPRT (*T. gondii*-ΔHXGPRT [41]) and introduced the *CpIMPDH* gene under the control of a *T. gondii* promoter [14]. Next the native *T. gondii IMPDH* gene was disrupted by replacing the entire coding sequence with a chloramphenicol acetyl transferase cassette using a new cosmid-based gene targeting approach [34]. The recombineering procedure used to modify a cosmid of the IMPDH genomic locus is summarized in Figure S1. Successful disruption of the gene was confirmed by PCR (data not shown) and Southern blotting (Figure 2A & B); note that numerous previous attempts to target this locus using smaller plasmid-based constructs failed. This manipulation created strain *T. gondii*-*Cp*IMPDH-ΔHXGPRT-Δ*Tg*IMPDH. Lastly, we introduced a fluorescent protein cassette and isolated stable transgenic parasites by cell sorting. The resulting strain is referred to as *T. gondii*-*Cp*IMPDH. The growth of this new parasite can be conveniently monitored in live cultures in 96 or 384 well format using a fluorescence plate reader [37]. We similarly engineered fluorescent versions of wild-type *T. gondii* and *T. gondii*-ΔHXGPRT to serve as controls.

### Pharmacological validation of the *Toxoplasma* reporter parasite

**Figure 3:**
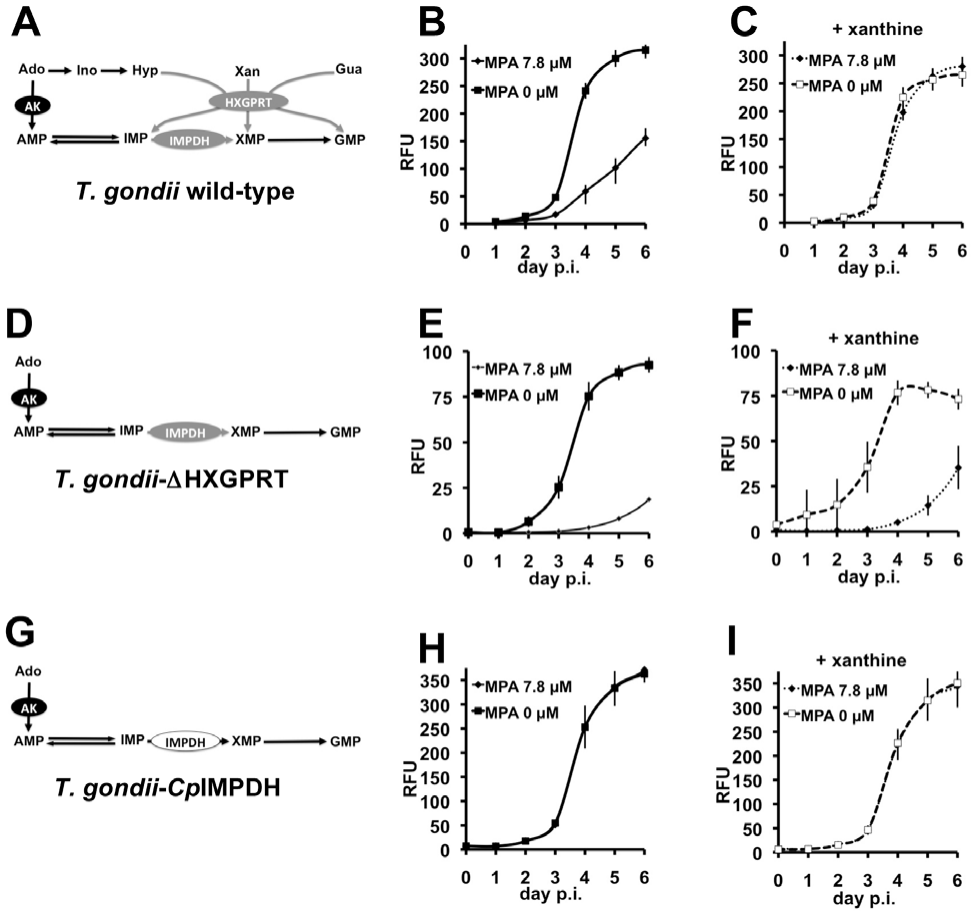
Validation of the *T. gondii-Cp*IMPDH reporter parasite. Schematic representation of the routes to GMP for the wild-type *T. gondii*, *T. gondii*-ΔHXGPRT, and *T. gondii-Cp*IMPDH are shown in **A**, **D** & **G** respectively. **B**, **E** & **H** show parasite growth in the presence of 0 µM and 7.8 µM MPA for wild-type *T. gondii*, *T. gondii*-ΔHXGPRT, and *T. gondii-Cp*IMPDH respectively. **C**, **F**, and **I** show parasite growth curves in the presence of 0 µM and 7.8 µM MPA, with the addition of 0.33 mM xanthine to the culture media, for wild-type *T. gondii*, *T. gondii*-ΔHXGPRT, and *T. gondii-Cp*IMPDH respectively. Data are representative of 2 independent experiments. Abbreviations as in Figure 1.

To determine if the proliferation of *T. gondii*-*Cp*IMPDH depends on *Cp*IMPDH as designed, we performed fluorescence growth assays in the presence of varying concentrations of mycophenolic acid (MPA), comparing wild-type *T. gondii*, ΔHXGPRT, and *Cp*IMPDH parasites (Figure 3A, D &G). MPA is a potent inhibitor of eukaryotic IMPDHs including *Tg*IMPDH [17], [42] but a very poor inhibitor of prokaryotic IMPDHs. *Cp*IMDPH is of prokaryotic origin and not inhibited by MPA at concentrations active against *Tg*IMPDH [14], [15]. As predicted, both wild-type *T. gondii* and *T. gondii*-ΔHXGPRT are sensitive to MPA (Figure 3B & E; [13], [14], [42]), but *T. gondii*-*Cp*IMPDH is resistant (Figure 3H). *T. gondii*-ΔHXGPRT is the most sensitive strain due to its inability to salvage xanthine/guanine from the media (EC_50_ = 0.29 µM versus 2.6 µM for the wild-type strain; Figure S2 details how an EC_50_ value was derived from the parasite growth curves). Supplementation of the media with xanthine (0.33 mM) essentially renders wild-type *T. gondii* MPA resistant (Figure 3C; EC_50_≥78 µM), but has no effect on *T. gondii*-ΔHXGPRT (Figure 3F). In contrast, *T. gondii*-*Cp*IMPDH is resistant to MPA in the absence of xanthine (EC_50_>65 µM), as expected given the resistance of the prokaryotic *Cp*IMPDH (Figure 3G & I; [15]).

These results demonstrate that the *T. gondii* model system provides a powerful tool for the evaluation of *in vivo* efficacy, selectivity, and specificity of *Cp*IMPDH inhibitors. Compounds that selectively inhibit *Cp*IMPDH will block the proliferation of *T. gondii*-*Cp*IMPDH but not the wild-type and *T. gondii*-ΔHXGPRT strains that depend on an eukaryotic IMPDH much like the human host. In contrast, non-specific compounds that have off-target activities in the parasite or the host cell will inhibit the growth of all three strains. A general inhibitor of both prokaryotic and eukaryotic IMPDHs will block the proliferation of both *T. gondii*-*Cp*IMPDH and *T. gondii*-ΔHXGPRT in the presence of xanthine, but will have no effect on the wild-type strain which contains HGXPRT (note that such compounds should be detected in our enzyme assays and eliminated before they reach this screen). Lastly, compounds showing poor efficacy against the *T. gondii*-*Cp*IMPDH parasite may signal problems pertaining to compound uptake, stability or metabolism. Examples of these varied outcomes are discussed below.

### A high-content imaging assay to evaluate anti-cryptosporidial activity of compounds

**Figure 4:**
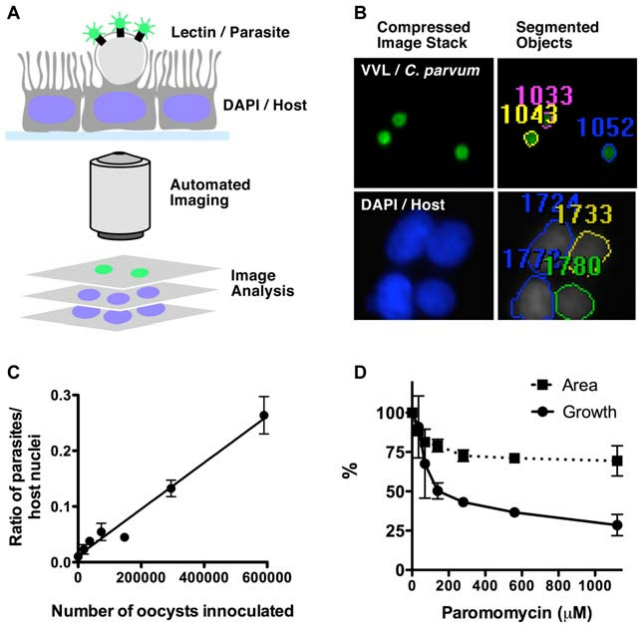
Overview and validation of the high content imaging *C. parvum* growth assay. **A**, schematic representation of differential labelling of parasite and host. **B**, detail of an exemplary micrograph obtained through the screening routine. Numbers indicate object identifies after segmentation analysis. **C** shows a 2-fold titration of *C. parvum* oocysts where the top concentration was 5.9×10^5^ oocysts per well. For **D**, the ratio of the number of FITC-VVL labelled parasites to DAPI labelled host cell nuclei was used to standardize each well. Percent *C. parvum* growth (solid line) was normalized to parasites receiving DMSO alone. The paromomycin EC_50_ for *C. parvum* growth was 97 µM. Paromomycin in addition to reducing parasite number also reduces the average size of the parasite (dashed line). Mean parasite area was measured for each treatment in triplicate. The percent area was then calculated respective to the mean area of parasites receiving DMSO alone. Data shows the mean of two independent experiments set up with triplicate wells in a 96-well format.

While the *Toxoplasma* model provides outstanding throughput and an excellent first filter it does not model all aspects of *Cryptosporidium* biology. A direct and efficient assay of *Cryptosporidium* proliferation was also needed. Fluorescent *Vicia villosa* lectin (VVL) has been used previously to score *C. parvum* growth by fluorescence microscopy [43]–[45]. VVL binds with high specificity to the *C. parvum* parasite, labelling sporozoites, the inner oocyst wall, and most importantly intracellular stages [40], [44], [45] but not the outer oocyst wall [46]. To accommodate the increasing number of compounds entering the SAR pipeline, we adapted the FITC-VVL immunofluorescence assay to a 96-well plate format and developed an automated imaging and analysis pipeline. Figure 4 shows an overview of the methodology. Plates are fixed, permeabilized and stained with FITC-VVL and DAPI to enumerate parasites and host cells, respectively. Using a spinning disc high-content microscope a 1.08 mm^2^ area of each well, was imaged providing a robust sample typically consisting of ∼6000 host cells and ∼2000 parasite stages. The instrument is programmed to automatically move from well to well, focus and acquire 20 µm deep image stacks for the entire plate. A series of automated image compression, manipulation, and object-finding algorithms was optimized for the recognition of host cells and parasites using the DAPI and FITC channels respectively (Figure 4 A and B, see Materials and Methods for further detail). To normalize for background staining and biological variability, control wells are included for background subtraction. The massive data output is stored, managed and accessed through an Accelrys pipeline database that performs further statistical analyses and transforms raw counts into percentage growth relative to a “no drug” control. Figure 4C shows a 2-fold titration of oocysts where the highest inoculum was 5.9×10^5^ oocysts per well, for *C. parvum* growth assays 5×10^5^ oocysts were added per well. Importantly, the algorithm can easily distinguish host and parasite, so false positives are not an issue.

The inhibition of parasite proliferation by paromomycin was evaluated to demonstrate the validity of this assay. The EC_50_ for paromomycin measured with this assay was 97 µM (Figure 4D), which is in good agreement with several previous studies (reported EC_50_ values range from 65–130 µM [20], [29], [47], [48]). Perplexingly, a significant number of parasites are observed even at very high concentrations of paromomycin (∼30% relative to the “no drug” control; Figure 4A). These residual parasites may be a developmentally arrested subset of the initial inoculum (note that a similar number of parasites is detected already after 24 hours, Figure S3). Interestingly, paromomycin was also found to significantly reduce the mean parasite area in a dose responsive manner (Figure 4D), which may also suggest arrested development.

### Validation of the fluorescent host cell growth assay

The differentiation of selective antiparasitic effects from those that are a secondary consequence of a host cell effect is a critical issue in drug discovery for intracellular parasites. Cytotoxicity assays commonly reflect plasma membrane integrity, and are a crude measure of host cell effects- it is conceivable that more subtle perturbations of host cell metabolism can have an adverse effect on parasite proliferation. Therefore we sought to develop a simple and inexpensive assay of host cell growth. The human ileocecal adenocarcinoma epithelial cell line, HCT-8, which is commonly used to maintain *C. parvum* infection in tissue culture, was engineered to constitutively express a green fluorescent protein (GFP). Growth of this cell line was monitored daily using a fluorescent plate reader. In agreement with previous reports [49]–[51], paromomycin had negligible effects on the growth of these cells (Figure S4A). The control sodium butyrate did inhibit HCT-8 growth in a dose-dependent manner in this assay (Figure S4B), as anticipated due to previous reports of its apoptotic effects in colonic tumor cell lines [52]. These experiments validate the use of the fluorescent HCT-8 cell growth assay.

### Identification of highly selective *Cp*IMPDH inhibitors in the *T. gondii* model

**Figure 5:**
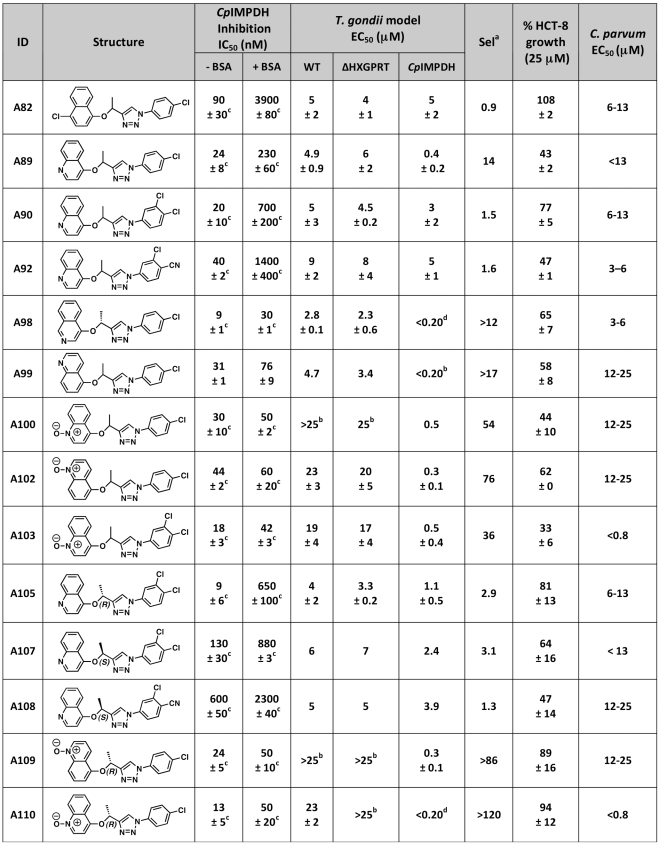
Compound structures and summary of activities. N.A., not applicable; N.D., not determined; a. Selectivity = EC_50_(*T. gondii*-wild-type)/EC_50_(*T. gondii-Cp*IMPDH); b. Highest concentration tested; c. Synthesis described previous study [22]; d. Lowest concentration tested; e. Determined in earlier study [20]; f. Determined using qPCR as described in [20]. Note that the structure and activities of additional amide derivatives of compound A are shown in Figure S5.

**Figure 6:**
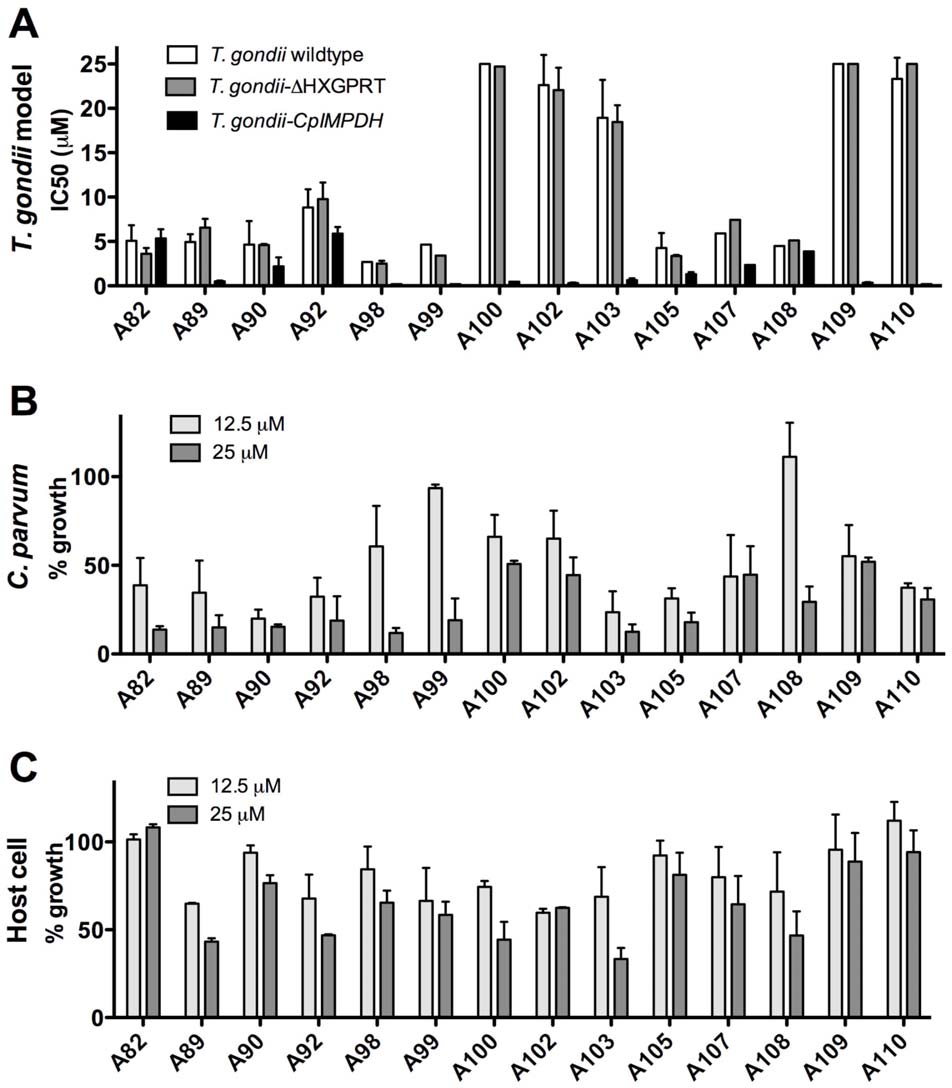
Identification of derivatives with high potency and selectivity in the *T. gondii-Cp*IMPDH model. **A** shows EC_50_ values for a selection of inhibitors assayed in the *T.gondii-Cp*IMPDH parasite model and demonstrates a range in inhibitor selectivity and potency. Compounds were assayed in triplicate and growth inhibition was calculated on a day during the exponential phase of growth, by normalisation to wells receiving DMSO alone. The EC_50_ calculation was performed as described in Figure S2. Compounds A82, A89, A90, A92, A102, A103, A105, and A110 were selected for rescreening and the mean values for at least 2 replicate experiments are shown. The inhibitors were then tested for inhibition of *C. parvum* (**B**) and host cell growth (**C**). For **B** percent *C. parvum* growth was determined using the high-content imaging assay as detailed in Figure 4, with the inhibitor at 12.5 µM and 25 µM. A subset of compounds was selected for re-screening and the mean over at least 2 replicate experiments is shown for compounds **A90**, **A92**, **A98**, **A103**, **A105**, **A109** and **A110**. **C** shows percent host cell growth assayed using the fluorescent HCT-8 cell line with inhibitors at 12.5 µM and 25 µM. GFP expressing HCT-8 cells were seeded at 4000 cells per well into 96-well plates and triplicate wells were spiked with test compound and fluorescence was measured daily for 7 days. Percent growth inhibition was calculated on a day during the exponential phase of growth, by normalisation to wells receiving DMSO alone. Inhibitors A89, A90 and A92 were selected for re-screening and the mean over at least 2 replicate experiments is shown.

The antiparasitic activities of compounds from our medicinal chemistry optimization program of compound A (identified in our initial screen [20]) were evaluated in the *T. gondii* model system. The structures of these compounds are shown in Figure 5 (triazoles) and Figure S5 (amides) alongside a summary of findings. Three compounds, A30, A57 and A66, do not inhibit *Cp*IMPDH *in vitro*; as expected, none of these compounds selectively blocked the growth of *T. gondii*-*Cp*IMPDH (Figures S5 & S6A). The remaining compounds inhibit *Cp*IMPDH with values of IC_50_ ranging from 9 nM to 2.6 µM [22]. Figure 6A shows representative data for fourteen 1,2,3-triazole derivatives in the *T. gondii* model; the remaining compounds are shown in Figure S6A. With the exception of A23 and A31, all compounds inhibit the growth of the *T. gondii*-*Cp*IMPDH parasite with an EC_50_<10 µM (Figures 6B & S6). Five of the 1,2,3-triazole derivatives, A100, A102, A103, A109 and A110, exhibit selectivity ≥36-fold for the *T. gondii*-*Cp*IMPDH parasite over wild-type *T. gondii* (Figure 5). Therefore the antiparasitic effects of these compounds can be confidently attributed to the inhibition of *Cp*IMPDH. A99 is ≥17-fold selective and the remaining compounds range in selectivity from 0.9–14-fold (Figure 5). These compounds have similar effects on both wild-type and *T. gondii*-ΔHXGPRT parasites (Figure 5), indicating that the low selectivity derives from off-target effects unrelated to *Tg*IMPDH.

**Figure 7:**
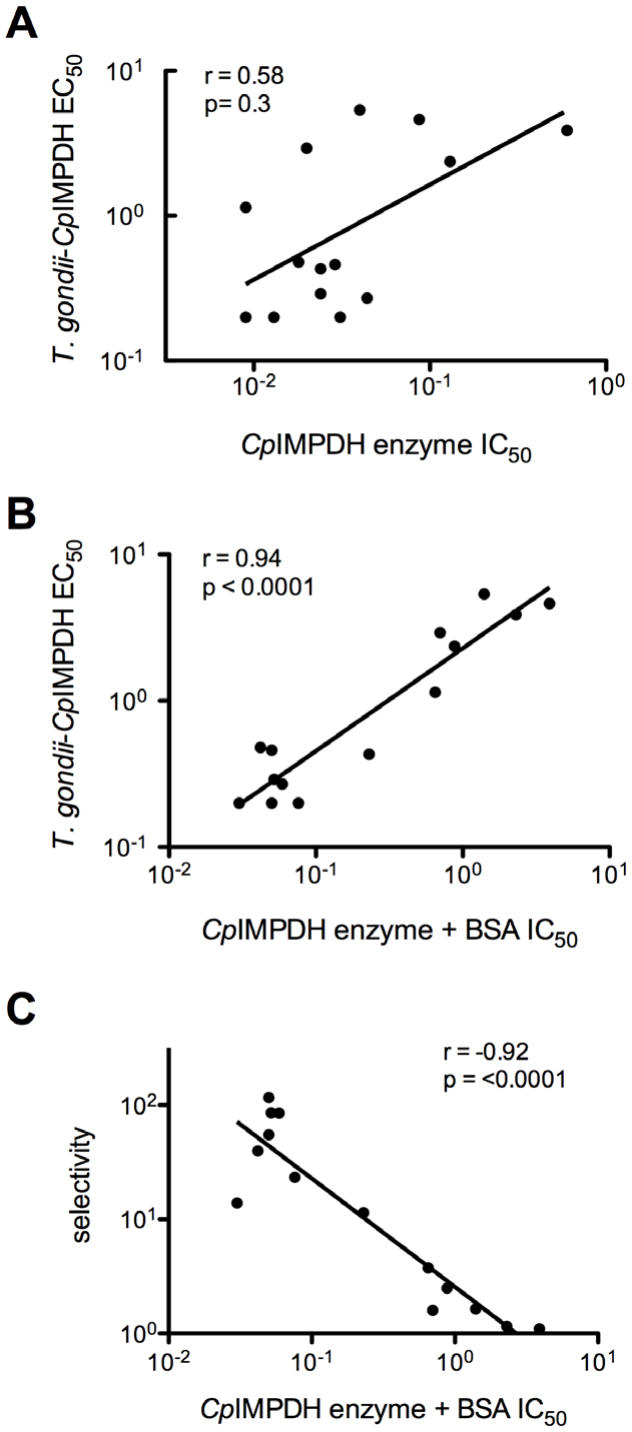
Correlation between *Cp*IMPH enzyme inhibition and potency and selectivity in the *T. gondii-Cp*IMPDH model. **A** shows a relatively weak (r = 0.58) and statistically insignificant (p-value = 0.3) correlation between the triazole compound IC_50_ values for the *Cp*IMPDH enzyme inhibition and the EC_50_ for proliferation of the *T. gondii-Cp*IMPDH parasite. However, a strong, positive correlation exists between the potency of *Cp*IMPDH enzyme inhibition when assayed in the presence of BSA and inhibition of *T. gondii*-*Cp*IMPDH proliferation (r = 0.94, p<0.0001; **B**). **C**, shows that selectivity in the *T. gondii* model, determined by the relative inhibition of the *T. gondii*-*Cp*IMPDH parasite over wild-type *T. gondii*, also correlates well with the potency of enzyme inhibition in the presence of BSA (r = −0.92, p<0.0001).

Surprisingly, initially we did not observe a significant correlation between the potency of a compound in the enzyme assay and inhibition of *T. gondii*-*Cp*IMPDH proliferation (Figure 7A). We wondered if some compounds might be binding nonspecifically to other proteins. To test this hypothesis, *Cp*IMPDH inhibition was also evaluated in the presence of BSA [22]. A strong positive correlation is observed between inhibition of *T. gondii*-*Cp*IMPDH proliferation and potency of enzyme inhibition in the presence of BSA (Figure 7B; r = 0.94, p<0.0001). Selectivity in the *T. gondii* model also correlates well with the potency of enzyme inhibition in the presence of BSA (Figure 7C; r = −0.92, p<0.0001). These observations indicate that the IC_50_ value in the presence of BSA is a useful proxy for antiparasitic activity modelled by *T. gondii*-*Cp*IMPDH proliferation.

### The *T. gondii* model predicts off-target host cell effects

Host cell growth was also assayed to assess the contribution of host cell effects to antiparasitic activity (Figures 6C and S6B). In general, strong host cell effects are observed in compounds that display little selectivity in the *T. gondii* model (Figures 5 & S5). With few exceptions, compounds that inhibited the proliferation of wild-type *T. gondii* with EC_50_≤10 µM also inhibited the proliferation of host cells. Compounds A82, A90 and A105 have values of EC_50_≤10 µM for all three *Toxoplasma* strains, and do not inhibit host cell growth, suggesting that their antiparasitic activities do not result from the inhibition of *Cp*IMPDH or *Tg*IMPDH. Instead, these compounds may act on other *T. gondii* targets not present in the host cell. Conversely, A100, A102, and A103 have EC_50_s of ≥20 µM against wild-type *T. gondii* yet inhibit HCT-8 cell growth significantly at 12.5 µM and 25 µM (Figure 5).

### The anti-cryptosporidial activities of *Cp*IMPDH inhibitors

The high-content imaging assay was used to evaluate the anti-cryptosporidial activity of the 1,2,3-triazole *Cp*IMPDH inhibitors at 12.5 µM and 25 µM (Figure 6B). All compounds inhibited *C. parvum* growth by at least 48% at a concentration of 25 µM (Figure 6B) and thus had equal or markedly improved anticryptosporidial efficacy when compared to parent compound A [20]. Unlike paromomycin, a significant reduction in parasite area was not detected (data not shown). The average area of the host cell nucleus was also recorded as a potential indicator of host cell cytotoxicity and likewise no significant change in host cell nuclei size was detected (data not shown). Encouragingly there was a negative trend between anticryptosporidial activity and host cell growth inhibition (data not shown), indicating that improvements in anticryptosporidial activity are not coincident with secondary effects on the host cell.

**Figure 8:**
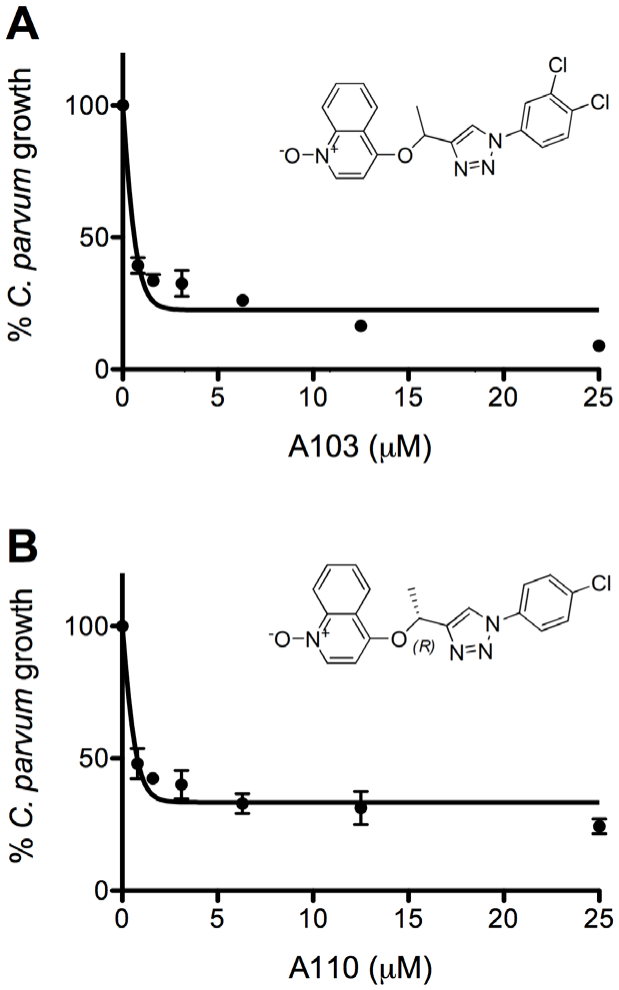
Compounds A103 and A110 are potent inhibitors of *C. parvum* growth. *C. parvum* growth was determined using the HCI assay. The ratio of the number of FITC-VVL labelled *C. parvum* parasites to DAPI labelled HCT-8 host cell nuclei was used to standardize each well and percent *C. parvum* growth was normalised to parasites receiving DMSO alone. **A** and **B** show compounds A103 and A110 respectively (EC_50_<0.8 µM). Data shows the mean of two independent experiments with triplicate wells.

To provide quantitative data to the SAR pipeline, the values of EC_50_ were determined for seven compounds. A82, A90, A92, A98 and A105 had EC_50_ values between 3 µM and 13 µM (Figure 5). Compounds A103 and A110 were found to be potent inhibitors of *C. parvum* growth with EC_50_ values of <0.8 µM (Figure 8A & B). As shown above, A103 and A110 also have negligible effects on host cell growth and exhibit good selectivity in the *T. gondii* model. Therefore, A103 and A110 have improved specificity and efficacy, and are promising candidates for advancement into mouse models of infection.

While not every compound that showed activity against the *T. gondii*-*Cp*IMPDH parasite had strong anticryptosporidial activity, none of the compounds showing poor activity in the *T. gondii*-*Cp*IMPDH model display significant anticryptosporidial activity. The *T. gondii* assay also immediately flagged compounds with poor permeability or off-target effects. We conclude that the *T. gondii*-*Cp*IMPDH model provides valuable information regarding antiparasitic activity and is a fast and highly informative filter for compound progression through medicinal chemistry optimization.

### Conclusions

Despite the tremendous public health impact of cryptosporidiosis, efforts to develop new and more effective treatments for this disease have been languishing. There are a number of reasons for this, but lack of suitable tissue culture and animal models to assess drug candidates is currently the most prominent roadblock. To overcome this challenge, we have developed a facile screening pipeline to evaluate the antiparasitic activity of *Cp*IMPDH inhibitors. The backbone of this pipeline is provided by a *T. gondii* model parasite that mirrors *Cryptosporidium* purine nucleotide pathways and depends on *Cp*IMPDH. The *T. gondii* model reliably eliminates compounds from further consideration and provides a useful filter to identify off-target activities. However, efficacy in the *T. gondii* model does not always guarantee anti-cryptosporidial activity. This disparity likely arises from differences in the biology of the two parasites. *T. gondii* and *C. parvum* infect different tissues, and occupy different intracellular compartments. The parasitophorous membrane of *T. gondii* is in direct contact with the host cell cytoplasm [53]. In contrast, *C. parvum* remains beneath the apical membrane of the host cell and is considered ‘extracytoplasmic’ due to the presence of a parasite induced host cell actin patch along with other peculiar and still largely uncharacterized structures, including the feeder organelle and a dense band visible in electron micrographs [54], [55]. These structures separate the parasite's parasitophorous vacuole from the host cell cytoplasm [56] and have been hypothesized to be involved in drug and nutrient uptake [57]. Furthermore, the two parasites, and their respective host cells, have different repertoires of drug efflux transporters [58], which may also account for the differences in inhibitor sensitivity. While the *T. gondii* assay does not fully negate the necessity of testing in *Cryptosporidium* directly, it has proven indispensible to winnowing candidate compounds to a number amenable to this more challenging model. We have used the pipeline to identify two promising candidates for anticryptosporidial chemotherapy: A103 and A110. These compounds are >100× more potent than paromomycin, the current standard for anticryptosporidial activity.

## Supporting Information

Figure S1 **Schematic overview of TOXOU05 cosmid recombineering.** Open triangles show positions of 50 bp gene specific primer sequences used to guide recombination in *E. coli*. See [34] for additional technical detail and reference.
(0.22 MB TIF)

Figure S2 **Obtaining an EC_50_ value for** ***T. gondii*** **growth.** Fluorescent *T. gondii* parasites are seeded into 96-well plates and spiked with test compound. Fluorescence is measured daily with a SpectraMax M22/M2e (Molecular Devices) plate reader for 6–7 days. The fluorescence readings on a day during the exponential phase of the growth curve, for example day 4 in **A**, are used to calculated percent growth inhibition. These values are fitted using the 4 parameter model y = D+(A–D)/(1+(x/C)^B^) where D is the minimum value, A is the maximum value, C is the EC_50_ and B is the Hill coefficient, using the SoftMax Pro v5 software, as illustrated in **B**. The absolute EC_50_ is recorded at the x intercept where y = 50.
(0.33 MB TIF)

Figure S3 **Time-course of VVL** ***C. parvum*** **growth assay.** HCT-8 cells were infected with *C. parvum* oocysts and the ratio of parasites to host cell nuclei was measured using the VVL assays as detailed in Fig. 4 after 1 or 2 days. Wells were either left uninfected (squares), were infected and treated with paramomycin (circles), or infected and treated with a DMSO solvent control (triangles).
(0.15 MB TIF)

Figure S4 **Validation of the HCT-8 pmaxGFP host cell growth assay.** HCT-8 cells constitutively expressing GFP seeded at 4000 cells per well into 96-well plates and triplicate wells were spiked with test compound. Fluorescence was measured daily with a SpectraMax M22/M2e (Molecular Devices) plate reader (Ex 485, Em 530) for 7 days. **A** shows a titration of paromomycin and **B** sodium butyrate.
(0.27 MB TIF)

Figure S5 **Compound structures and summary of activities.** N.A., not applicable; N.D., not determined; a. Selectivity = EC_50_(*T. gondii*-wild-type)/EC_50_(*T. gondii*-*Cp*IMPDH); b. Highest concentration tested; c. Synthesis described previous study [22]; d. Lowest concentration tested; e. Determined in earlier study [20]; f. Determined using qPCR as described in [20].
(5.26 MB TIF)

Figure S6 **Antiparasitic activity of the A series in the** ***T. gondii*** **model of** ***Cryptosporidium*** **infection.** **A** shows the EC_50_ for a selection of compounds assayed in the *T.gondii-Cp*IMPDH parasite model. Compounds were assayed in triplicate and growth inhibition was calculated on a day during the exponential phase of growth, by normalisation to wells receiving DMSO alone. The EC_50_ calculation was performed as described in Figure S2. Note the highest concentration tested in panel A was for compound A30 was 20 µM. **B** shows percent host cell growth assayed using the GFP fluorescent HCT-8 cell line with compound at 25 µM and 50 µM.
(0.97 MB TIF)

Supplemental Materials S1 NMR spectra data for compounds A61, A64, A68 and A99.
(0.03 MB DOC)
