# Supplementary figures and images for: A Screening Pipeline for Antiparasitic Agents Targeting Cryptosporidium Inosine Monophosphate Dehydrogenase
Source: PLoS Negl Trop Dis. 2010 Aug 10;4(8):e794. doi: 10.1371/journal.pntd.0000794 (PMC2919388; doi:10.1371/journal.pntd.0000794)

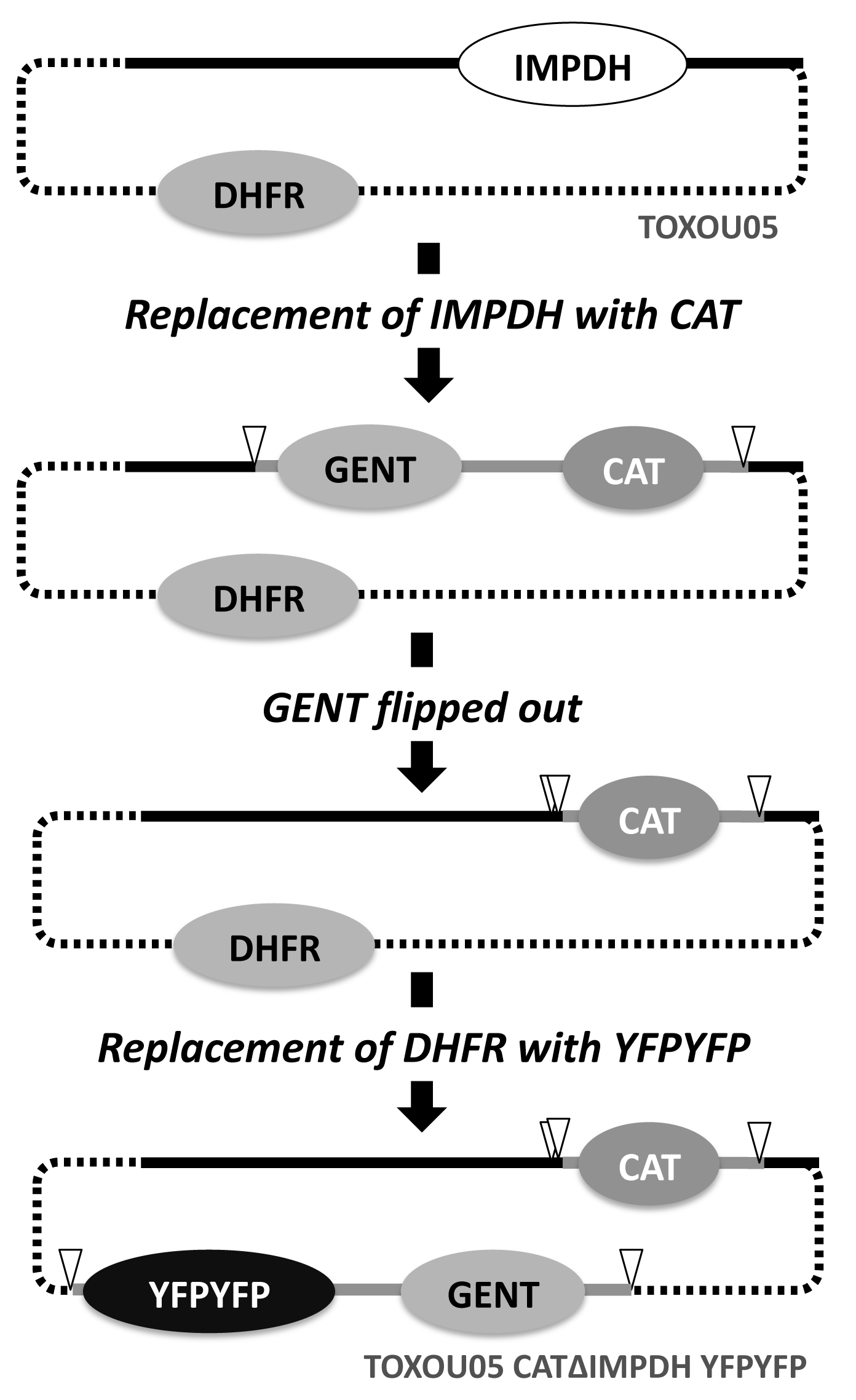

Supplement: Figure S1 — Schematic overview of TOXOU05 cosmid recombineering. Open triangles show positions of 50 bp gene specific primer sequences used to guide recombination in E. coli. See [34] for additional technical detail and reference. (0.22 MB TIF) [file pntd.0000794.s001.tif]

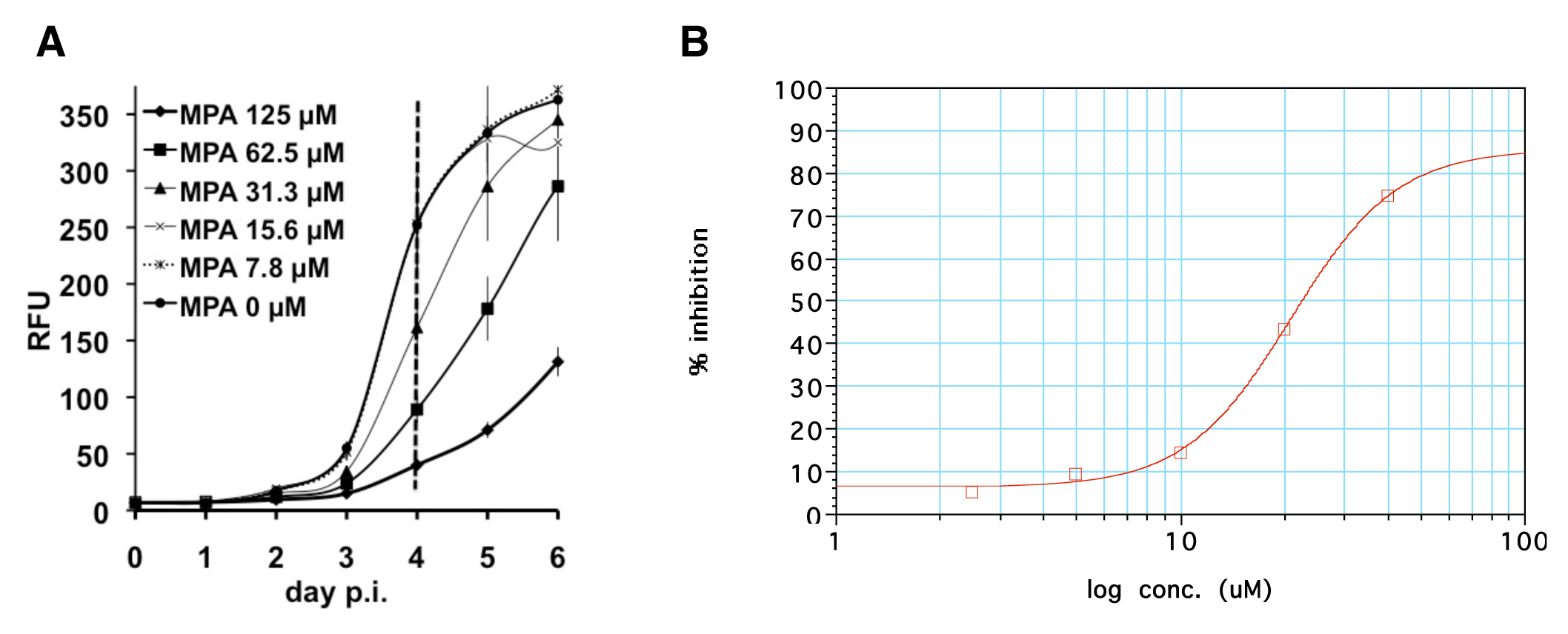

Supplement: Figure S2 — Obtaining an EC50 value for T. gondii growth. Fluorescent T. gondii parasites are seeded into 96-well plates and spiked with test compound. Fluorescence is measured daily with a SpectraMax M22/M2e (Molecular Devices) plate reader for 6–7 days. The fluorescence readings on a day during the exponential phase of the growth curve, for example day 4 in A, are used to calculated percent growth inhibition. These values are fitted using the 4 parameter model y = D+(A–D)/(1+(x/C)B) where D is the minimum value, A is the maximum value, C is the EC50 and B is the Hill coefficient, using the SoftMax Pro v5 software, as illustrated in B. The absolute EC50 is recorded at the x intercept where y = 50. (0.33 MB TIF) [file pntd.0000794.s002.tif]

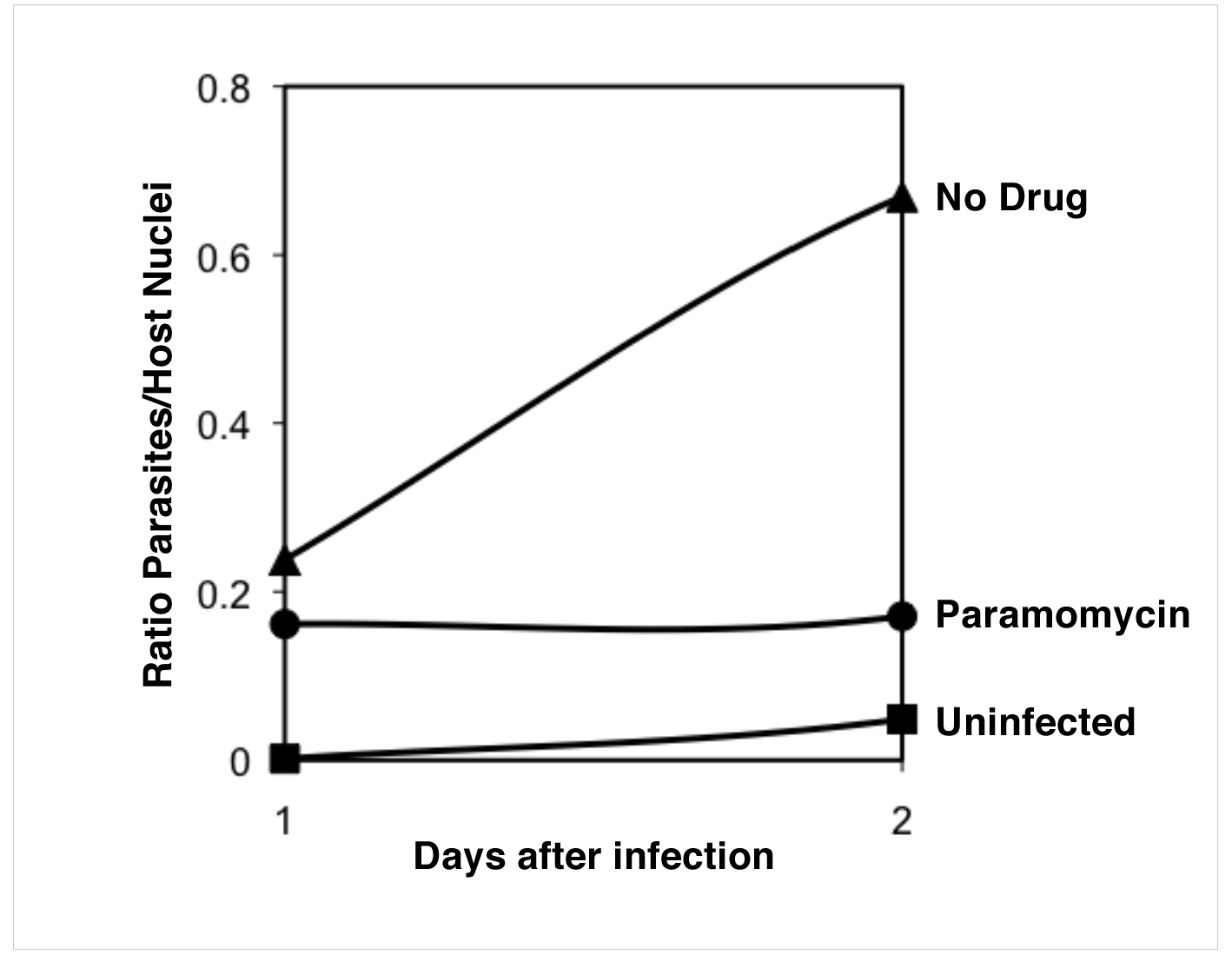

Supplement: Figure S3 — Time-course of VVL C. parvum growth assay. HCT-8 cells were infected with C. parvum oocysts and the ratio of parasites to host cell nuclei was measured using the VVL assays as detailed in Fig. 4 after 1 or 2 days. Wells were either left uninfected (squares), were infected and treated with paramomycin (circles), or infected and treated with a DMSO solvent control (triangles). (0.15 MB TIF) [file pntd.0000794.s003.tif]

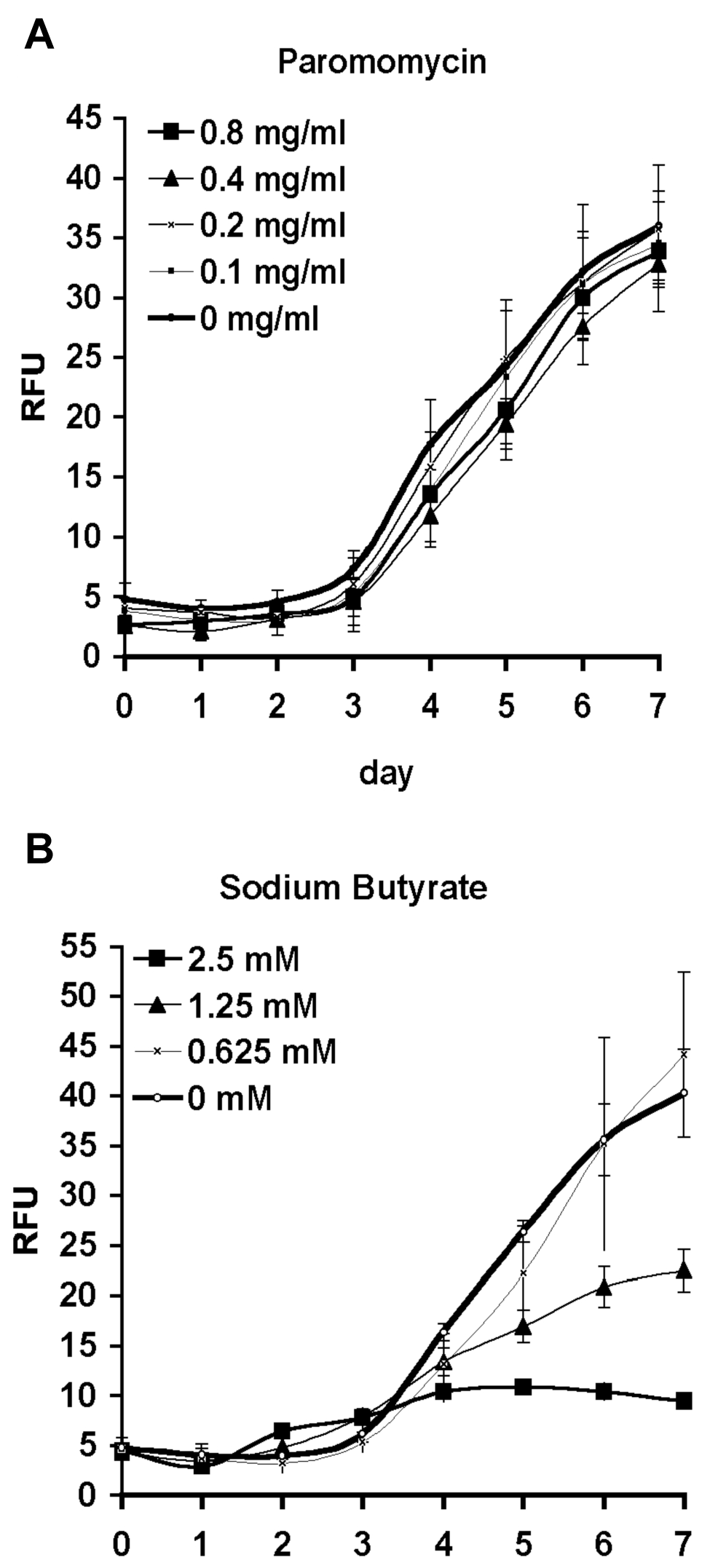

Supplement: Figure S4 — Validation of the HCT-8 pmaxGFP host cell growth assay. HCT-8 cells constitutively expressing GFP seeded at 4000 cells per well into 96-well plates and triplicate wells were spiked with test compound. Fluorescence was measured daily with a SpectraMax M22/M2e (Molecular Devices) plate reader (Ex 485, Em 530) for 7 days. A shows a titration of paromomycin and B sodium butyrate. (0.27 MB TIF) [file pntd.0000794.s004.tif]

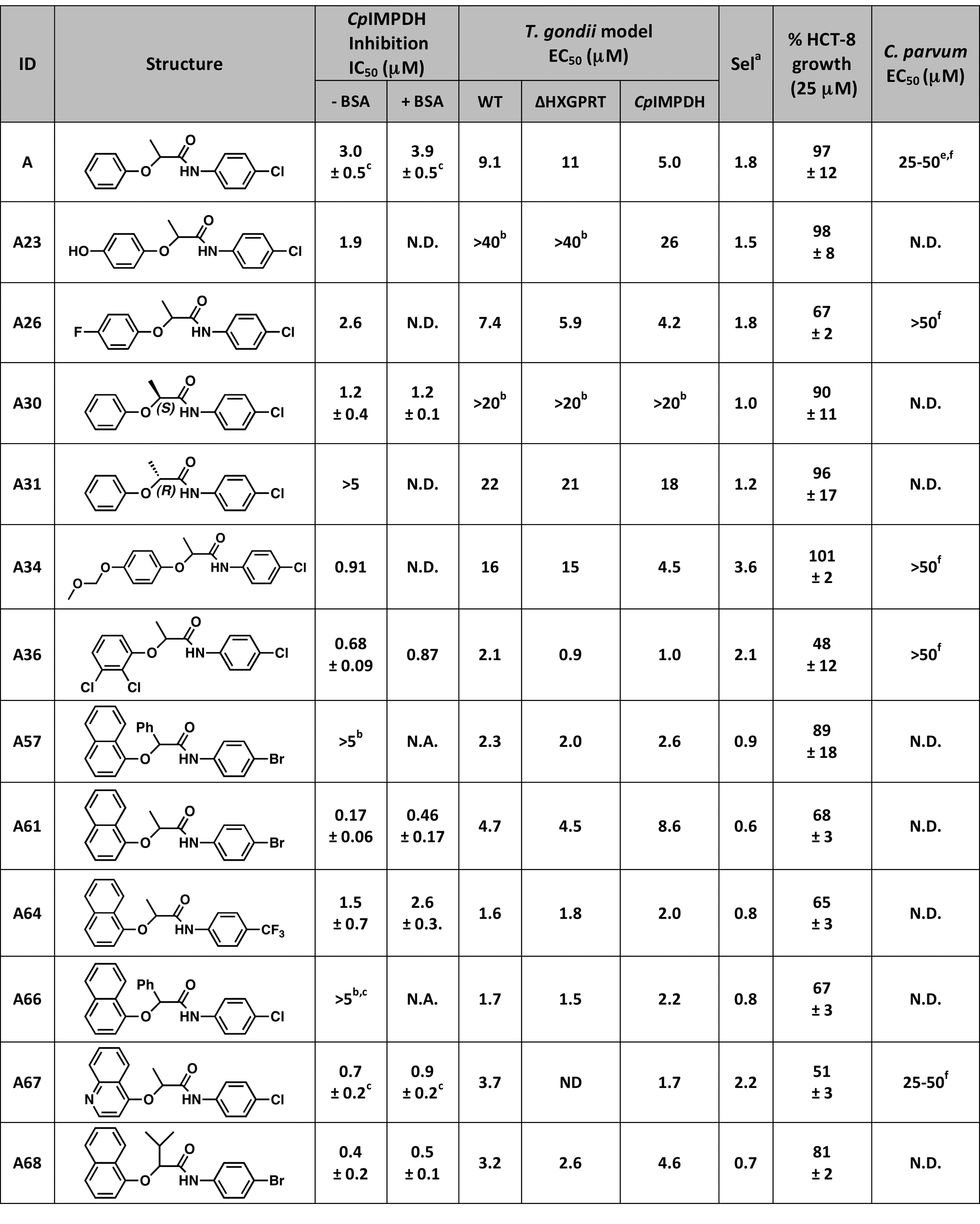

Supplement: Figure S5 — Compound structures and summary of activities. N.A., not applicable; N.D., not determined; a. Selectivity = EC50(T. gondii-wild-type)/EC50(T. gondii-CpIMPDH); b. Highest concentration tested; c. Synthesis described previous study [22]; d. Lowest concentration tested; e. Determined in earlier study [20]; f. Determined using qPCR as described in [20]. (5.26 MB TIF) [file pntd.0000794.s005.tif]

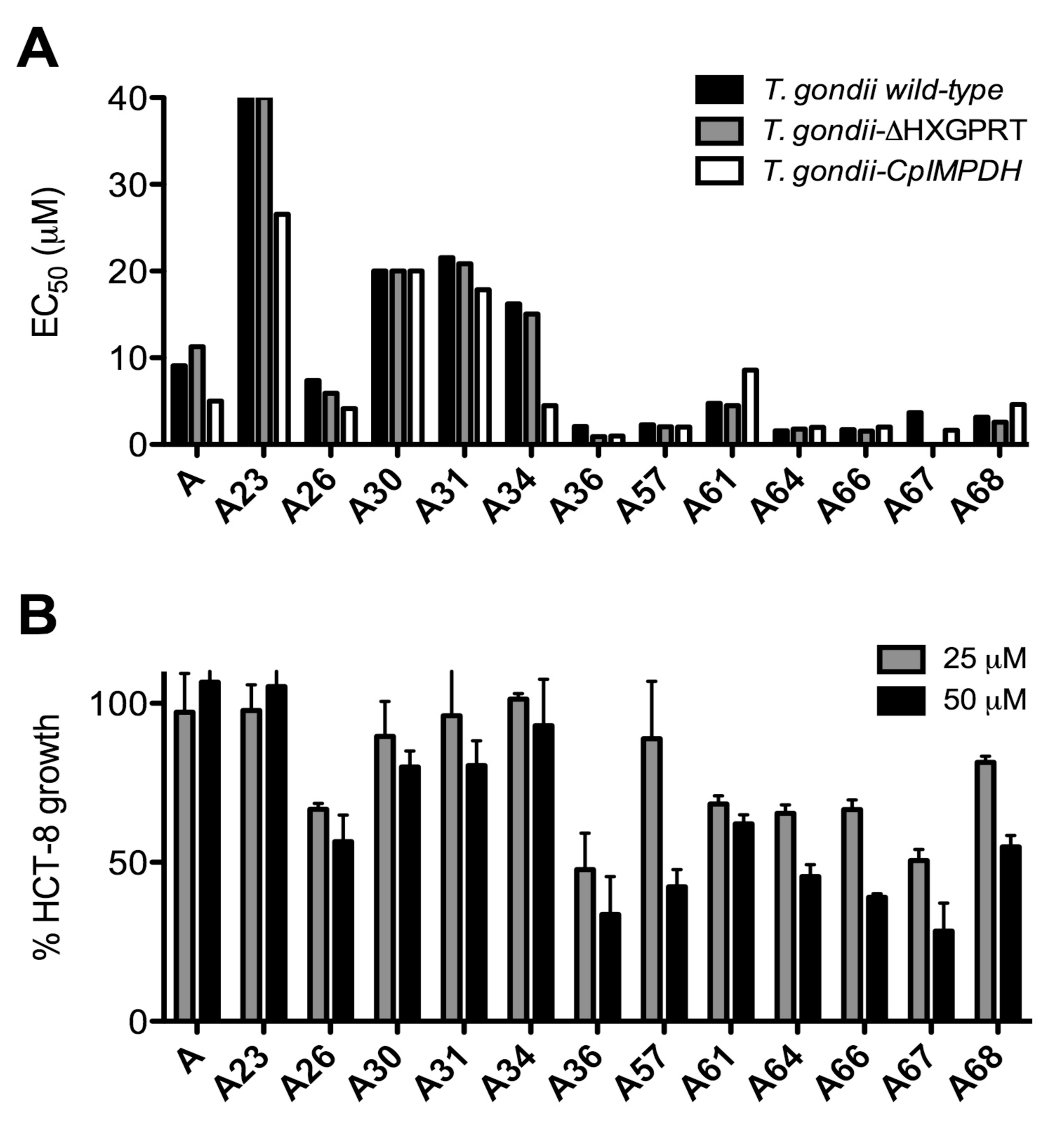

Supplement: Figure S6 — Antiparasitic activity of the A series in the T. gondii model of Cryptosporidium infection. A shows the EC50 for a selection of compounds assayed in the T.gondii-CpIMPDH parasite model. Compounds were assayed in triplicate and growth inhibition was calculated on a day during the exponential phase of growth, by normalisation to wells receiving DMSO alone. The EC50 calculation was performed as described in Figure S2. Note the highest concentration tested in panel A was for compound A30 was 20 µM. B shows percent host cell growth assayed using the GFP fluorescent HCT-8 cell line with compound at 25 µM and 50 µM. (0.97 MB TIF) [file pntd.0000794.s006.tif]
