# Supplementary material for: A Screening Pipeline for Antiparasitic Agents Targeting Cryptosporidium Inosine Monophosphate Dehydrogenase
Source: PLoS Negl Trop Dis. 2010 Aug 10;4(8):e794. doi: 10.1371/journal.pntd.0000794 (PMC2919388; doi:10.1371/journal.pntd.0000794)
Supplement: Supplemental Materials S1 — NMR spectra data for compounds A61, A64, A68 and A99. (0.03 MB DOC) [file pntd.0000794.s007.doc]

**Supplemental Material S1**

**NMR spectra data for A61, A64, A68 and A99.**

*N*-(4-bromophenyl)-2-(1-naphthalenyloxy)propanamide (**A61):** ^1^H NMR (CDCl_3_, 400MHz): δ 1.79 (d, *J* = 6.8 Hz, 3H), 4.98 (q, *J* = 6.8 Hz, 1H), 6.87 (d, *J* = 7.6 Hz, 1H), 7.37 (t, *J* = 8.4 Hz, 1H), 7.42 (s, 4H), 7.52-7.59 (m, 2H), 7.85-7.87 (m, 1H), 8.23(bs, 1H), 8.29-8.32 (m, 1H).

2-(naphthalen-1-yloxy)-N-(4-(trifluoromethyl)phenyl)propanamide (**A64**): ^1^H NMR (CDCl_3_, 400MHz): δ 1.79 (d, *J* = 6.8 Hz, 3H), 4.98 (q, *J* = 6.8, 13.2 Hz, 1H), 6.87 (d, *J* = 7.6 Hz, 1H), 7.37 (t, *J* = 8.4 Hz, 1H), 7.53-7.58 (m, 4H), 7.64 (d, J = 8.4 Hz, 2H), 7.85-7.88 (m, 1H), 8.30-8.39 (m, 2H).

*N*-(4-Bromoophenyl)-3-methyl-2-(1-naphthalenyloxy)butanamide (**A68**)**:** yield 91 %; ^1^H NMR (CDCl_3_, 400 MHz) δ 1.22 (dd, *J*_1_ = 22.8 *J*_2_ = 6.8, Hz, 6H), 2.54 (m, 1H), 4.68 (d, *J* = 4.4 Hz, 1H), 6.83 (d, *J* = 8.0 Hz, 1H), 7.25 (m, 2H), 7.35 (t, *J* = 8.0 Hz, 1H), 7.40 (d, *J* = 8.8 Hz, 2H), 7.52 (d, *J* = 8.0 Hz, 1H), 7.54-7.60 (m, 2H), 7.86 (m, 1H), 8.01 (s, 1H), 8.38 (m, 1H); ^1^

5-(1-(1-(4-chlorophenyl)-1H-1,2,3-triazol-4-yl)ethoxy)quinoline (**A99**)**:** yield 82 %; mp. 94-96 ^o^C; ^1^H NMR (CDCl_3_, 400 MHz) δ 1.92 (d, *J* = 6.4 Hz, 3H), 5.89 (q, *J* = 6.8 Hz, 1H), 7.00 (d, *J* = 8.0 Hz, 1H), 7.40 (dd, *J*_1_ = 8.0 Hz, *J*_2_ = 3.6, 1H), 7.45 (t, *J* = 2.4 Hz, 1H), 7.47 (t, *J* = 2.4 Hz, 1H), 7.55 (t, *J* = 8.4 Hz, 1H), 7.63 (t, *J* = 2.0 Hz, 1H), 7.65 (t, *J* = 2.0 Hz, 1H), 7.69 (d, *J* = 8.8 Hz, 1H), 7.88 (s, 1H), 8.65 (d, *J* = 8.0 Hz, 1H), 8.91 (dd, *J*_1_ = 4 Hz, *J*_2_ = 2 Hz, 1H);
